# Dynamic interplay: disentangling the temporal variability of fish effects on coral recruitment

**DOI:** 10.1038/s41598-023-47758-6

**Published:** 2023-11-28

**Authors:** Jamie M. McDevitt-Irwin, Douglas J. McCauley, Daniel R. Brumbaugh, Franziska Elmer, Francesco Ferretti, Timothy D. White, Joseph G. Wible, Fiorenza Micheli

**Affiliations:** 1 Hopkins Marine Station, Stanford University, Pacific Grove, CA USA; 2 Marine Science Institute, University of California, Santa Barbara, CA USA; 3 Department of Ecology, Evolution, and Marine Biology, University of California, Santa Barbara, CA USA; 4 Elkhorn Slough National Estuarine Research Reserve, Watsonville, CA USA; 5 Environmental Studies, University of California, Santa Cruz, Santa Cruz, CA USA; 6 School for Biological Sciences, Victoria University of Wellington, Wellington, New Zealand; 7 Center for Marine Resource Studies, School for Field Studies, Cockburn Harbour, South Caicos Turks and Caicos Islands; 8 Fish and Wildlife Conservation Department, Virginia Tech, Blacksburg, VA USA; 9 Center for Ocean Solutions, Stanford University, Pacific Grove, CA USA

**Keywords:** Community ecology, Coral reefs

## Abstract

Ecosystems around the world are continuously undergoing recovery from anthropogenic disturbances like climate change, overexploitation, and habitat destruction. Coral reefs are a prime example of a threatened ecosystem and coral recruitment is a critical component of reef recovery from disturbances. Reef fishes structure this recruitment by directly consuming macroalgae and coral recruits or by indirectly altering the substrate to facilitate coral settlement (e.g., grazing scars). However, how these direct and indirect mechanisms vary through time remains largely unknown. Here, we quantified coral recruitment on settlement tiles with divots that mimic grazing scars and caging treatments to exclude or allow fish feeding over 3 years at Palmyra Atoll in the Pacific Ocean. We found that the positive and negative effects of fishes on coral recruitment varies through time. After 3 years, both grazing scars and fish grazing no longer predicted coral recruitment, suggesting that the role of fishes decreases over time. Our results emphasize that reef fish populations are important in promoting initial coral recovery after disturbances. However, over time, factors like the environment may become more important. Future work should continue to explore how the strength and direction of top-down control by consumers varies through time across multiple ecosystems.

## Introduction

As populations and ecosystems around the world are being threatened by a range of escalating stressors from climate change, including temperature warming and extreme weather events, it is imperative to increase our understanding of recovery after these disturbances. Coral reefs are an illustrative example of an ecosystem under threat^1–4^. Coral recruitment, an integral facet to reef recovery, is impacted by factors including available substrate^5,6^, micro-habitats/surface irregularity^7–9^, competition with algae^10,11^ and predation^12,13^, and benthic feeding fishes such as corallivores and herbivores (i.e., families in Labridae, Scaridae, Pomacentridae, Chaetodontidae and Acanthuridae). These fishes can consume macroalgae that compete with corals, directly or incidentally consume coral recruits, or provide clean substrate through bite scars^14–17^. However, fish populations are also changing globally, due to fishing pressure, habitat alterations, and other disturbances^18,19^. Increased knowledge is needed to understand the role of fishes in shaping coral recruitment to improve predictions of future consequences and management of the continued pressures in tropical coastal ecosystems.

Benthic feeding fishes influence coral recruitment and survival through direct and indirect pathways. Herbivorous fishes can indirectly promote coral recruitment by consuming competitive macroalgae, which in turn, creates space for less palatable crustose coralline algae (CCA), which can increase coral recruitment and survival^10,15,17,20–22^. However, both herbivores and corallivores can negatively affect coral recruitment through direct or accidental predation^23–26^. Herbivorous fish are usually grouped within four functional groups based on their feeding strategy- scrapers, excavators, grazers and browsers^27^. Parrotfishes, a unique group of herbivorous fishes with beak-like jaws that bite and scrape or excavate the surface of the benthos, can indirectly affect coral recruitment by physically modifying the substrate with bite marks. The size of these bite scars varies with fish body size^28,29^. For example, a large parrotfish species like *Bolbometopon muricatum* would leave a larger scar than a smaller species like *Chlorurus microrhinos.* This unique feeding mode can remove all benthic organisms and leave a clear scar on the substratum, thus potentially influencing successional processes on the reef by opening up new settlement space and restarting the successional clock^14,30^ that may create a “recruitment window” for coral recruits^31^.

These bite marks may also act as microhabitats or refuges for coral settlers from predation, by creating heterogeneity and three-dimensional structure which could promote coral recruitment^8,9,32–35^. Microhabitats can act as refuges for coral settlers from predation^8,9,32,34,35^ and grooves based on parrotfish bite marks have been previously shown to promote coral recruitment^33^. However, the role of microhabitats can depend on fish grazing; a previous experiment found coral recruitment was 9X higher in protected crevices in the uncaged than the caged treatments^23^. Yet nearly all studies have been limited at approximately 1 year in duration or less and have been conducted in regions that have historically or currently face high fishing pressure^16,17,21,24,36,37^ . Therefore, it remains relatively unexplored how the role of fishes in structuring coral recruitment may vary through successional time, especially at a site with relatively intact consumer assemblages and intense grazing pressure.

Here, we evaluate how benthic feeding fishes influence patterns of coral recruitment at Palmyra Atoll, a remote and uninhabited wildlife reserve in the Pacific Ocean. Using 180 caged (to exclude large fishes) and uncaged settlement tiles with custom divots to mimic parrotfish grazing scars, we monitored coral recruitment at 2 weeks, 1 year and 3 years after tile deployment. This study site is unique due to its relatively intact food web due to no local fishing pressure, with high fish and shark biomass^38–40^. This fish community includes large terminal phase parrotfishes and large species such as *Bolbometopon muricatum,* thus providing a potentially strong contrast for the caging treatment. We ask the questions: (1) Do benthic feeding fishes influence patterns of coral recruitment? (2) Do these effects, if present, occur directly through grazing and/or indirectly through grazing scars? And (3) How do these impacts vary through time?

## Results

Two weeks after tile deployment, we found a total of 1791 potential coral recruits with an average of 9.43 recruits per tile for the caged treatment and 14.1 recruits for the uncaged treatment. After 1 year, we had a total of 193 coral recruits with an average of 1.4 recruits on the caged tiles and 0.82 recruits on the uncaged tiles. However, after 3 years, there were only 60 coral recruits with an average of 0.52 recruits on the caged tiles and 0.37 recruits on the uncaged tiles. 1 and 3 years after tile deployment, the highest number of coral recruits was observed on the edges of tiles, rather than on the flat surfaces or in the divots of the tiles. Of the coral recruits we could identify visually; we detected the genus *Pocillopora, Porites and Acropora*, with the majority within *Pocillopora* and *Porites*, which are the two most abundant coral genera on the forereef of Palmyra^41^.

**Figure 1:**
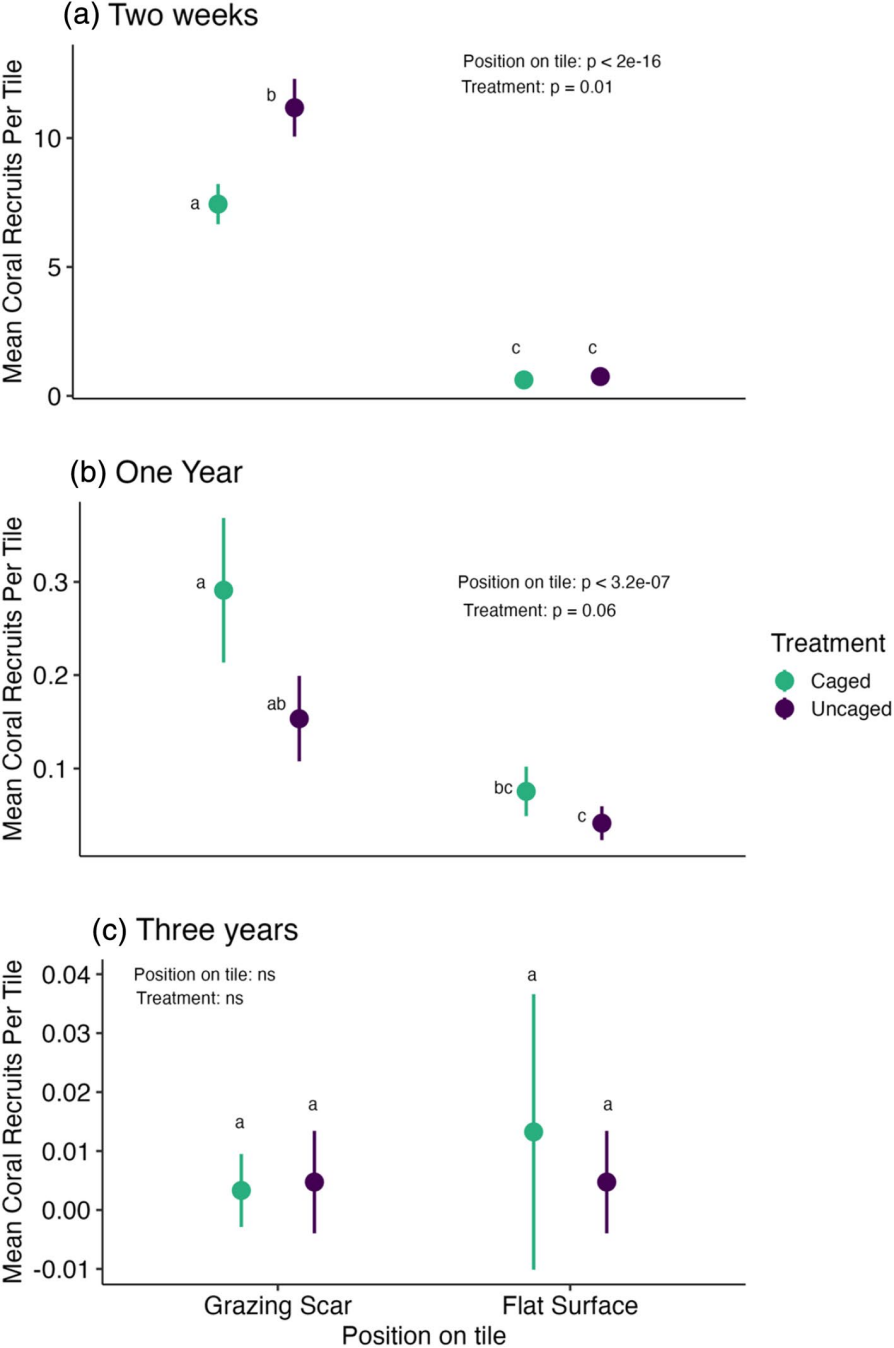
Least square means (+ /- SE) of coral recruits per tile for position on the tile (grazing scars vs flat surface) and treatment (caged vs uncaged, green and purple respectively) averaged across all sites for (**a**) 2 weeks, (**b**) 1 year and (**c**) 3 years after tile deployment. The letters (**a**, **b**, **c**) denote significant differences between the means. Note the different y-axes for each year.

Two weeks after tile deployment, position on the tile (X^2^ = 510.21, *df* = 1, *p* < 2e−16) and caging treatment (X^2^ = 6.28, *df* = 1, *p* = 0.01) were both significant drivers of coral recruitment (R^2^ Conditional = 0.86, R^2^ Marginal = 0.71, Fig. 1, Supplementary Table S1, Figure S7). However, position on the tile and caging treatment did not significantly interact (Supplementary Table S1). In both the caged and uncaged treatments, the divots had significantly higher coral recruit abundances than flat surfaces (Caged: t-ratio = 14.95, *p* < 0.0001; Uncaged: t-ratio = 17.51, *p* < 0.0001, Fig. 1, Figure S7). In fact, 93% of the coral recruits were found in the divots. Within the divots, the uncaged tiles always had higher coral recruit abundance than the caged tiles (t-ratio = − 3.76, *p* = 0.0012, Fig. 1, Figure S7). As reported above, flat surfaces had very low coral recruit abundances on both caged and uncaged tiles and with no significant differences between them (Fig. 1, Figure S7).

After 1 year, position on the tile was still the primary driver of coral recruitment (X^2^ = 26.13, *df* = 1, *p* = 3.2e−07), and caging treatment showed a similar trend to the 2-week sampling date, though not statistically significant (X^2^ = 3.54, *df* = 1, *p* = 0.06) (R^2^ Conditional = 0.49, R^2^ Marginal = 0.15, Fig. 1, Supplementary Table S1). Again, position on the tile did not significantly interact with caging treatment. There were significantly more coral recruits in the divots than the flat surfaces for both the caged (t-ratio = 4.5, *p* = 0.0001) and uncaged treatments (t-ratio = 3.08, *p* = 0.01). In contrast, after 3 years, neither position on the tile or treatment were significant drivers of coral recruit abundance (R^2^ Conditional = NA, R^2^ Marginal = 0.13, Fig. 1).

**Figure 2:**
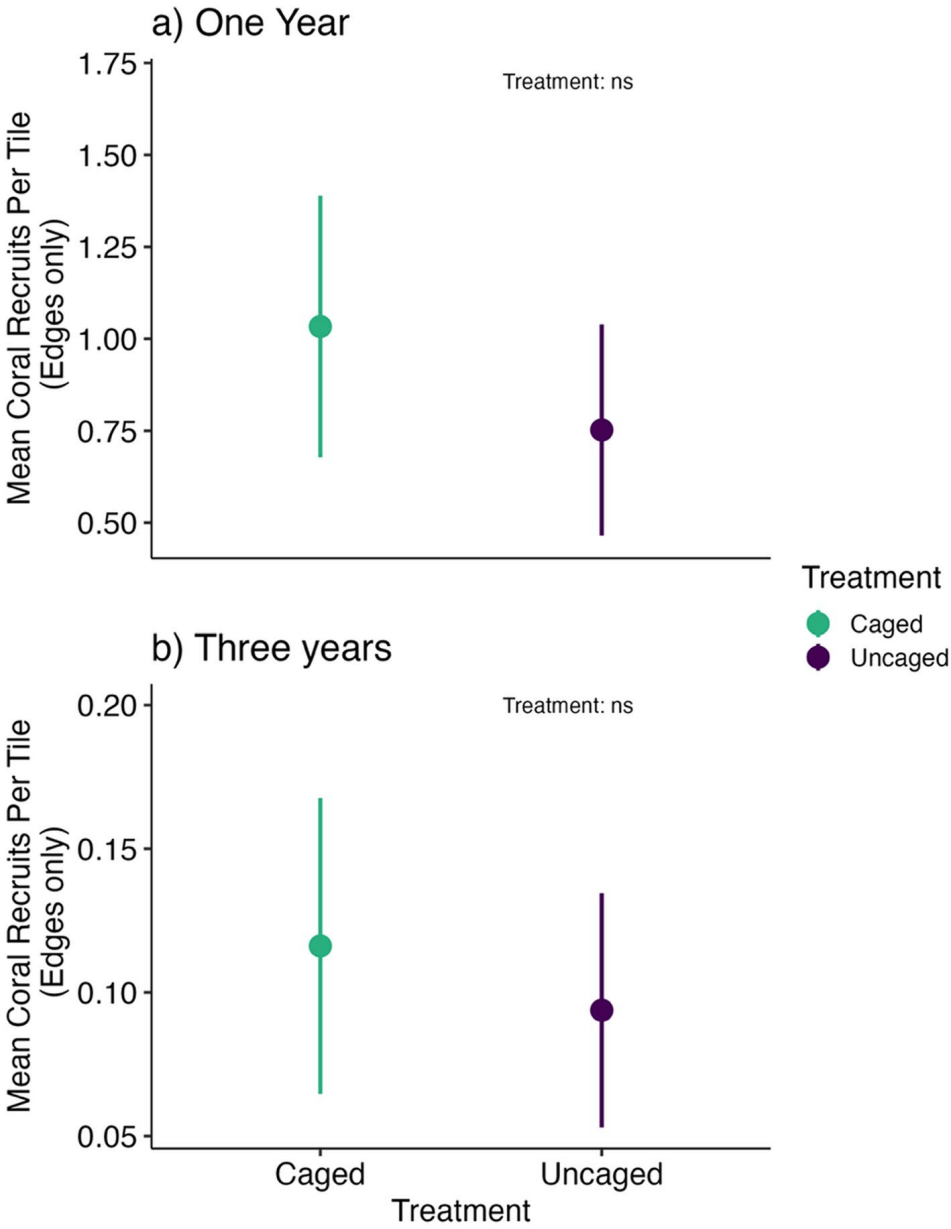
Least square means (+ /- SE) of coral recruits per tile (edges only) for each treatment averaged across all sites for (**a**) 1 year and (**b**) 3 years after tile deployment. Note the different y-axes for each year.

1 and 3 years after tile deployment, the highest number of coral recruits was observed on the edges of tiles, compared to the divots and the flat surfaces, with no significant effects of caging treatment on the abundance of coral recruits found on the edges of the settlement tiles (Fig. 2, Supplementary Table S2, Figure S8). 1 year after tile deployment, site (X^2^ = 6.67, *df* = 2, *p* = 0.04) was a significant predictor of coral recruit abundance (R^2^ Conditional = 0.54, R^2^ Marginal = 0.21), with FR9 having lower coral recruit abundance than FR3 and FR7 (Figure S9). 3 years after tile deployment, only site was a significant predictor of coral recruit abundance (X^2^ = 13.93, *df* = 2, *p* = 0.0009) (R^2^ Conditional = 0.51, R^2^ Marginal = 0.24, Fig. 2) with FR7 having the highest coral recruit abundance (Figure S9).

## Discussion

Overall, we found evidence that the positive and negative effects of benthic feeding fishes on coral recruitment, directly through grazing and indirectly through bite marks, varies through time. Divots mimicking grazing scars from parrotfishes were the primary driver of coral recruitment patterns and enhanced coral recruitment during early succession, suggesting these scars may act as important microhabitats for recruiting corals^8,9,32,34,35^, but these effects attenuated over 3 years. Initially, grazing by benthic feeding fish promoted coral recruitment, but after 1 year this trend reversed, with more corals found in the caged tiles rather than the uncaged tiles. These results suggest that initially, large fishes may promote coral recruitment, but after 1 year, direct predation by such fishes may surpass the positive effects of decreased competition and negatively impact coral recruits^16,23–25^. Similarly, the effect of caging decreased over time and was not significant 3 years after tile deployment. Thus, our results suggest that fishes structure short term patterns of coral recruitment through both grazing scars and direct grazing, but over the long term this top down control diminishes. Other factors like temperature stress^42,43^, the *Symbiodinium*^44,45^ /microbiome community^46,47^, or the other successional taxa^48^ may become more important drivers through successional time.

### Grazing scars from parrotfishes promote coral recruitment during early succession

Divots mimicking grazing scars from parrotfish promoted coral recruitment up to 1 year after the new substrate was available, potentially because they provided a refuge from accidental or direct predation. Similarly, microhabitats have been previously found to promote coral recruitment on settlement tiles^8,9,32–34^. However, contrary to our results Doropoulos et al.^23^ found that microhabitats interacted with the presence of fishes. They found the highest coral recruitment was in crevices in uncaged treatments, potentially because when fish are excluded, corals face competition with algae that may also use these microhabitats as a refuge^49^. However, we did not find statistical significance of caging treatment interacting with the divots. Potentially our divots, which are shallower (e.g. 0.3 and 0.4 cm vs. 1 cm) and wider (1.8 L and 3.6 L vs. 1.2 L cm) than Doropoulos et al.’s^23^ crevices, may not create such a highly competitive environment with algae (e.g. increased water flow to get rid of algae exudates like microbes and nutrients). Thus, reinforcing the idea that size^35^ and dimension may influence the role of microhabitats in driving coral recruitment patterns. Our results emphasize that not only do parrotfish play an important role in maintaining algal communities and bioerosion^50^, but also they create microhabitats with their grazing scars, thus promoting coral recruitment^33^.

### The impact of fish grazing on coral recruitment varies through time

Initially, excluding herbivores resulted in lower coral recruitment, but after 1 year, this relationship reversed and excluding herbivores promoted coral recruit abundance. This suggests that fish exposure initially promotes coral recruitment, but over time, direct or incidental predation may impact coral survival, thus emphasizing important tradeoffs during ontogeny^23^. This result suggests that competition with macroalgae may be a strong driver during early successional stages, whereas corallivory may become a dominant driver in later successional stages. The algae, *Lobophora,* declined in abundance in these caged tiles over time^51^ providing support for the hypothesis that coral-algae competition was stronger in year 1 than year 3. Furthermore, the community composition became more similar between the uncaged and caged tiles through time, with increases in *Lobophora* and declines in CCA on the uncaged tiles^51^ suggesting competition and/or facilitation would likely show larger differences between caging treatments in year 1 than year 3.

Exposure to fishes has been previously found to have both positive^10,17,21,22^ and negative^16,23–25^ impacts on coral recruitment. Caging can protect juvenile corals from predation and increase survival^12,52,53^, but excluding fishes can also increase the amount of macroalgae and decrease a coral facilitator, CCA^10,17,21,24^. The mixed positive and negative results from our study and previous literature, suggests that the role of fishes likely varies with coral ontogeny (as emphasized by Doropoulos et al.^23^), the environment, the pre-existing fish community, or reef degradation. For example, Palmyra is a highly remote and protected region, and the high fish abundance and grazing pressure may have a greater negative impact on coral recruits vs more disturbed regions like Hawaii^21^, the Caribbean^10,17^ and Palau^22^ that depend on fishes to control algal growth, especially in environmental contexts where expansion of algae is accelerated by anthropogenic nutrient pollution. Additionally, previous research has demonstrated that herbivore control of coral recruitment varies with herbivore composition. O’Leary et al.^37^ found that fish grazing promoted coral recruitment in marine protected areas dominated by herbivorous fishes, whereas on fished reefs with high abundances of sea urchins, sea urchin grazing reduced coral recruit survival. A previous study in Palmyra found that fish exclosures promoted coral recruitment after one month^25^ (the opposite of our 2 week results), potentially due to benthic feeding fishes causing significant coral mortality after one month. Therefore, survey timing may be integral when measuring coral recruitment tradeoffs, with differences between even 2 and 4 weeks. However, in agreement with our results, they also found that the role of fishes in structuring coral recruitment decreased over time (after four months). Our differences in methodology may contribute to these differences in results, for example, our tiles have divots and flat surfaces whereas their tiles only had flat surfaces.

Additionally, it’s important to note that we did not differentiate between survival and new settlement for year 1 and year 3. Initially, 2 weeks after tile deployment, there was high coral recruitment in both the uncaged tiles and in the grazing scars, however, this potentially did not translate to high coral survival over time. For example, corals exposed to fishes may have experienced high mortality from predation that led to no significant difference in coral recruitment between caged and uncaged after 3 years. Additionally, corals that initially recruited to divots, may have experienced mortality from competition with other sessile organisms like algae. Finally, recruitment levels could be so low 3 years after post-settlement mortality, that we may be unable to capture statistical differences in recruitment between caging treatments and tile position.

### The role of fishes decreased over time in driving coral recruitment patterns

After 3 years, both herbivore exposure and grazing scars no longer drove patterns of coral recruitment, suggesting that factors like environmental conditions (e.g. temperature stress), the *Symbiodinium*^44,45^ /microbiome community^46,47^, or the other successional taxa^48^ may become more important in driving coral success and survival over time. For example, these same caged tiles had more *Lobophora* and less CCA than the uncaged tiles^51^. *Lobophora* can negatively impact coral recruitment by releasing algae exudates^54^ and thus the rest of the successional community may play a more important role later in succession that overrides the initial short-term benefit of grazing scars or protection from predation. Additionally, after 3 years, the rest of the successional community may have filled in the divots with growth (e.g. paved in by CCA, turf, etc.) and caused the grazing scars to become less similar to microhabitats on the reef. Previous research has found similar short-term effects of fish grazing on coral recruitment, with these effects becoming insignificant after 70 days^24^ and four months^25^. These results are further supported by experimental work in protist communities that found top-down control by predators influenced prey community composition early in succession, but not at later stages in succession^55^. Our results emphasize recent calls for community ecology to more directly integrate temporal dynamics, as species interactions can vary with ontogeny, seasonality and climate change^56^.

### Vertical microhabitats promote coral recruitment

Coral recruits were consistently in highest abundance on the edge of the tile, in both year one and three. Thus, this vertical orientation may act as a type of microhabitat, similar to the grazing scars, that promotes coral recruitment. Previous research has demonstrated that vertical surfaces can promote juvenile coral abundance and survival^57^ potentially by reducing sedimentation and/or direct sunlight compared to horizontal surfaces. Additionally, research on coral reef algae communities has found that vertical surfaces are less affected by grazing and typically dominated by crustose coralline algae (CCA)^58^, thus, the high coral recruit abundance we observed at the edges of tiles may be driven by lower predation, increased settlement cues from CCA, or both. As coral reefs worldwide are at risk of losing structural complexity, from loss of live coral cover and shifts in the functional composition^59,60^, this potentially could have cascading consequences on coral recruitment as there could be less vertical substrate and microhabitats for coral settlement.

### Future research and limitations

Our results suggest that the role of benthic feeding fishes in influencing coral recruitment and recovery following disturbance is likely to vary over time, and may be more apparent during early succession, for example, immediately after disturbances like extreme heat waves from climate change^43,61,62^. As coral reefs may face recurring disturbances^63–65^, the impacts of losing fishes may become more important as coral reefs may continually be locked in early succession. Furthermore, small patches of the reef are regularly in an early successional stage, for example, from a coral dying or breaking from wave action. In addition, these impacts likely vary across reefs of different conditions and local pressures. Palmyra Atoll is a remote and protected system, with high fish biomass and low algae cover compared to more degraded reefs, and our results suggest that excluding large fishes from grazing may not play as important of a role in structuring coral recruitment as on reefs that have less fish and higher algae cover. Additionally, Palmyra Atoll may have higher coral recruitment than other more disturbed reefs with lower overall coral cover, thus on these reefs with lower recruitment, fishes may play a stronger role. Therefore, future research should consider how top-down control and tradeoffs vary across reefs with different degradation levels, fish communities, recruitment levels and benthic cover.

Our study is limited by the fact that we did not monitor individual coral survival or determine what was new recruitment at each time point. In addition, although our study is one of the longest evaluating the role of fishes in determining patterns of coral recruitment (3 years vs ~ 1 year typical of most studies), especially in a remote near-pristine reef, we were only able to monitor coral recruitment at three time points due to logistical limitations of visiting this remote atoll. Thus, we have a limited understanding of the fine scale temporal dynamics. Our 2-week time point would be more robust if we had taken the tiles back to the lab to identify coral recruits under the microscope, using the same methodology as year 1 and year 3, however, we were unable to use this methodology due to fieldwork logistics and time constraints. Furthermore, as we included site as a fixed effect instead of a random effect, this limits our generalizability of our results, however we found limited evidence for site being a significant driver of coral recruitment patterns (e.g., it was only significant in the edge model). Additionally, the flat surface of our tiles was slightly larger than our divot surface area (54 cm^2^ vs. 45 cm^2^) so there is a higher probability for recruits to settle on a flat surface, however our results show the exact opposite trend of higher recruitment in divots. Finally, although previous research has found that cages of this mesh size and in this system do not influence water flow or light attenuation^25^, our conclusions would be more robust if we had included our own cage control.

Finally, our use of completely bare, artificial tiles may have a slightly different effect than a grazing scar created on an already crowded and congested reef surface with competitors close by. The creation of bare space from a grazing scar may play a more significant role by opening bare space for coral recruitment. A previous study found that although corals recruited to settlement tiles, no new recruits were visible on natural surfaces, suggesting that reef recovery is likely influenced by available substrate and competition with other organisms^66^. Our patterns here may be influenced by the fact that over time, there was decreasing available space for new coral recruits and increased competition with other organisms for space.

## Conclusions

Our results suggest that fishes play an important role in determining the short-term patterns of coral recruitment, but over time their influence diminishes. Thus, our results suggest it is imperative to manage coral reef fish populations, especially herbivorous fishes like parrotfishes that not only eat competitive algae but also create bare substrate and microhabitats on the reef. This is especially important as coral reefs are likely increasingly locked in an early successional stage (e.g., after repeat bleaching events, storms/wave action) where fishes play an important role in structuring coral recruitment. Overall, our results demonstrate that the role of consumers in structuring recovery patterns likely varies through time both in strength and direction and should be tested in more ecosystems.

## Materials and methods

### Data collection

We conducted our field experiment at Palmyra Atoll, a remote and uninhabited wildlife refuge in the Pacific Ocean (5° 52’ N, 162° 04’ W, Figure S1), where fishing is prohibited within ~ 50 nautical miles of shore. As Palmyra Atoll is fully protected and remote, it provides a unique study system with nearly intact fish and shark food webs^38–40^ (Supplementary Fig. S5, S6). To evaluate how benthic feeding fishes influence coral recruitment, we used experimental settlement tiles. We deployed 180 10 × 10 cm custom unglazed ceramic settlement tiles, each with eleven divots that were hand engineered, two of them representing bumphead parrotfish (*Bolbometopon muricatum)* grazing scars (i.e. 3.7 L × 2.6 W × 0.4 D cm) and nine of them representing adult Pacific steephead parrotfish (*Chlorurus microrhinos)* grazing scars (i.e. 1.8 L × 1.1 W × 0.13 D cm) (Supplementary Fig. S3). The size of these divots was determined by calculating the average size of field measured divot bites delivered to the reef during focal follows of *B. muricatum* (n = 64) and *C. microrhinos* (n = 29). The divots took up 45 cm^2^ of the top surface area of the tile while 54 cm^2^ was flat surface.

Tiles were installed on a 15 × 15 cm high-density polyethylene baseplate and attached to the reef with a stainless-steel screw and anchor. The settlement tiles were deployed in pairs (within 1 m of each other) of a caged and uncaged tile (Supplementary Fig. S3). The cages were made of 5 × 5 cm mesh and have been previously shown to significantly reduce grazing by fishes larger than 10–15 cm (total length) and completely exclude fishes > 25 cm (total length)^25^. In our study, we did not include a cage control. However, previous research used this same mesh material in Palmyra and found that these cages did not significantly affect water flow or light entering the cage^25^. The tiles were deployed in July–August 2013 across three different sites (FR3: 5.86654 N, − 162.11359 W; FR7: 5.89715 N, − 162.07831 W; FR9: 5.89651 N, − 162.12813 W) and at three different average depths (~ 10.7 m, ~ 12.2 m, ~ 13.7 m) (Supplementary Fig. S1Two of these sites (FR9 and FR7) were placed on the north facing side of the atoll and the third (FR3) was selected on the south side to sample a range of different wave/wind exposure conditions and oceanographic variability found at Palmyra^67,68^.

The tiles were surveyed for coral recruits at three time points: 2 weeks after tile deployment, 1 year after deployment, and 3 years after deployment. During the two-week survey, we counted potential coral recruits in situ with underwater UV lighting^69,70^. This approach likely overestimates the number of coral recruits, as we counted anything that strongly fluoresced green. However, from personal observation using microscopic inspection during preliminary study of the 180 tiles over 3 years, stony corals dominate the proportion of tiny organisms that fluoresce bright green on the tiles, with anemones and octocorals estimated to make up less than 5%. This potential bias affects coral counts across all treatments because we are using the same methodology across both caged and uncaged tiles, enabling a relative comparison of recruit abundance among them. It is important to note, however, that results after two weeks may signal differences in recruitment of calcified invertebrates more broadly. For this reason, we did not compare coral recruit numbers between the two week and subsequent surveys, and we caution that one should not compare these coral recruitment rates over time, but rather between treatments. In years 1 and 3, we brought the tiles back to the lab and surveyed for recruits using a microscope and UV light. During transport from the field and analysis in the lab, tiles were stored without contacting one another in aerated seawater tanks. In year 1, tiles were then redeployed back on the reef in < 24 h to their exact location and orientation in the experiment. If a recruit was touching any part of a divot, it was counted as growing in that divot. Any coral touching the edge of the tile (even if also on the flat surface) was coded as growing on the edge. We did not differentiate between new and old recruitment on our tiles. Our definition of a “coral recruit” also includes juvenile corals that survived post-settlement processes (see Supplementary Fig. S4). We visually identified coral recruit images taken of tiles during the one- and three-year review to provide a qualitative assessment of coral diversity. Identification of corals was not possible during the initial 2-week review and a substantial fraction of corals could not be definitively identified from images alone. As such, analyses of recruitment were conducted with all coral genera pooled. A few tiles were lost during the experiment, or we could not survey them. As a result, the number of replicates varied among sampling dates: at the 2-week interval, we sampled 152 tiles in total, 177 tiles at 1 year, and 137 tiles at 3 years.

### Statistical analyses

We evaluated each year separately in the statistical models, as we were interested in the interaction between treatment (caged vs uncaged) and position on the tile (i.e., whether coral was in a divot vs on a flat surface), and we had survey methodology differences between some of our time points. We compared coral recruitment counts between treatments and position on the tile by including them as interacting fixed effects. We did not include site (FR3, FR7 vs FR9) as a random effect due to the limited number of levels, so we instead included it as a fixed effect. We included tile number nested within tile pairing as a random effect. We used generalized linear mixed-effect models within the “glmmTMB” package^71^. We fit each model with a Poisson, negative-binomial, zero-inflated Poisson, and zero-inflated negative binomial distribution and compared these models with AIC to determine the best model to use. For the two-week data, the best model fit was given by the negative binomial distribution. For the year 1 and 3 data, the best model fit was given by the Poisson distribution. We set sum-to-zero contrasts on each of our fixed effects since we planned to do a type 3 test. We then used a type 3 ANOVA in the car package^72^ to look at overall fixed effects, and then looked at the interaction between tile placement and treatment using the pairs() function in the eemeans package^73^. We checked model diagnostics (qqplot, residual vs predicted) using the DHARMa package^74^.

Many corals were found on the edge of the tile rather than the divots or flat surfaces. To examine recruitment patterns of these corals, we ran a separate model for how treatment (caged vs uncaged) influences corals found on the edges of the tiles. Corals recruited along the edge of tiles were only recorded in year 1 and 3. We included treatment and site as fixed effects and tile number nested within tile pairing as a random effect. We used generalized linear mixed-effect models from the glmmTMB package^71^ and used AIC to select between the distributions: Poisson, negative binomial, zero-inflated Poisson and zero-inflated negative binomial. For year 1, the best model to fit the data was a zero-inflated Poisson versus year 3 was a Poisson. We then used a type 2 ANOVA in the car package^72^ to look at overall fixed effects, and then conducted a post-hoc contrast for treatment using the pairs() function in the eemeans package^73^. Again, we checked model diagnostics (qqplot, residual vs predicted) using the DHARMa package^74^. For all analyses we used R Version 4.2.2. The data and code used for the statistical analyses are available at https://github.com/JamieMcDevittIrwin/McDevittIrwin_etal_PalmyraCoralRecruitment.

### Supplementary Information

Supplementary Information.

## Data Availability

The data and code used for the statistical analyses are available at https://github.com/JamieMcDevittIrwin/McDevittIrwin_etal_PalmyraCoralRecruitment.
