# Supplementary material for: Dynamic interplay: disentangling the temporal variability of fish effects on coral recruitment
Source: Sci Rep. 2023 Nov 28;13:20971. doi: 10.1038/s41598-023-47758-6 (PMC10684556; doi:10.1038/s41598-023-47758-6)
Supplement: Supplementary file 1 — Supplementary Information. [file 41598_2023_47758_MOESM1_ESM.pdf]

Supplementary Materials

Dynamic interplay: disentangling the temporal variability of fish effects on coral recruitment

Jamie M. McDevitt-Irwin[*1,2], Douglas J. McCauley[2,3], Daniel R. Brumbaugh[4,5], Franziska Elmer[6,7], Francesco Ferretti[8], Timothy D. White[1], Joseph G. Wible[1], Fiorenza Micheli[1,9]

*jamie.mcirwin@gmail.com

Table S1. Type 3 ANOVA tables on the generalized linear models of coral abundance.

Term	df	Chisq	p-value
Two-weeks			
Treatment	1	6.28	**0.01**
Position on the tile	1	510.21	**< 2e-16**
Site	2	3.62	0.16
Treatment * Position on the tile	1	0.95	0.33
Year 1			
Treatment	1	3.54	**0.06**
Position on the tile	1	26.13	**3.2e-07**
Site	2	3.40	0.18
Treatment * Position on the tile	1	0.005	0.94
Year 3			
Treatment	1	0.15	0.70
Position on the tile	1	1.49	0.22
Site	2	3.13	0.21
Treatment * Position on the tile	1	1.49	0.22

Table S2. Type 2 ANOVA tables on the generalized linear models for the corals found on the edge of the tiles.

Term	df	Chisq	p-value
Year 1			
Treatment	1	1.06	0.30
Site	2	6.67	**0.04**
Year 3			
Treatment	1	0.42	0.52
Site	2	13.93	**0.0009**

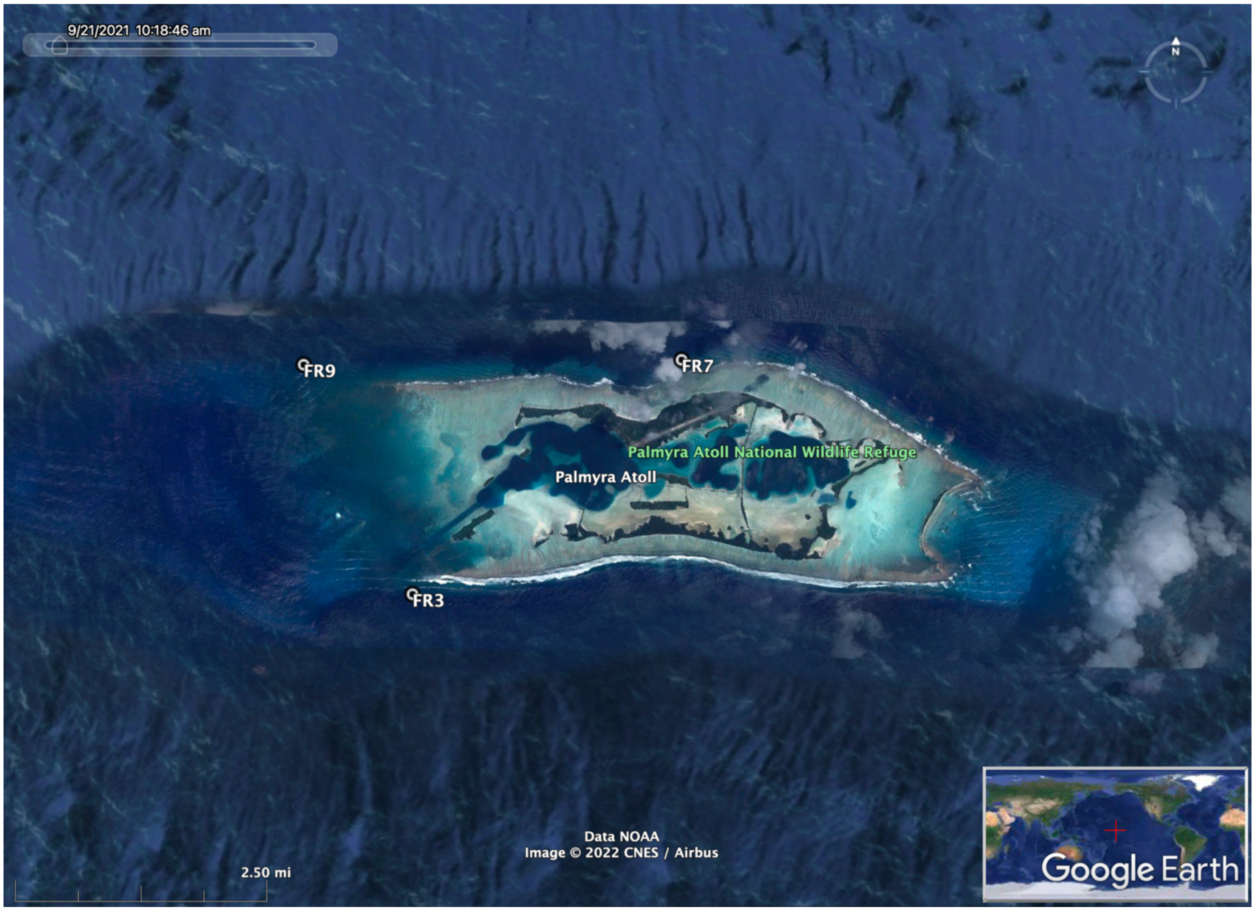

Fig. S1. Map of the study sites (FR3, FR7, FR9) at Palmyra Atoll in the Pacific Ocean.

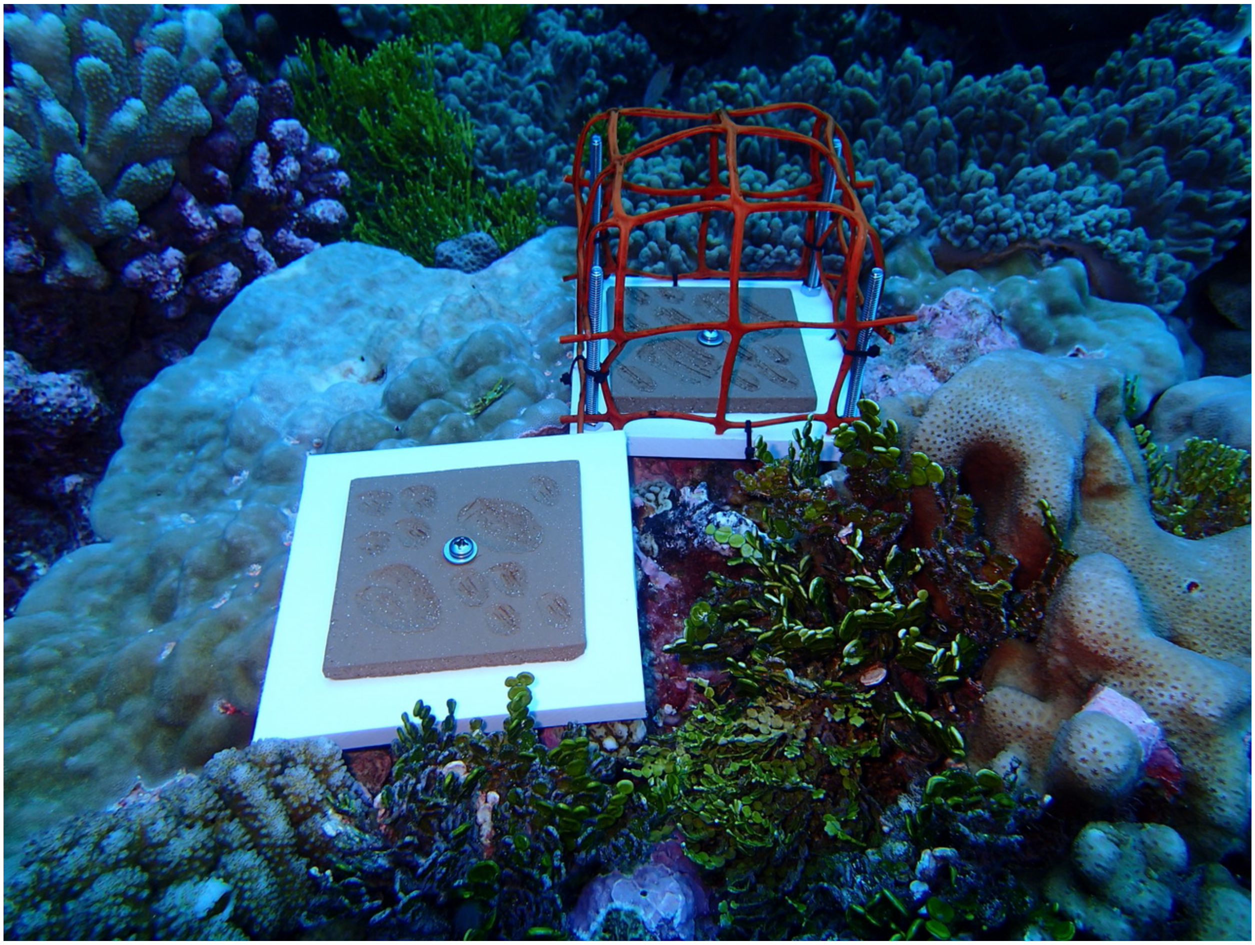

Fig. S2. Photo of an example pairing of caged and uncaged tiles (Photo Credit: Joe Wible).

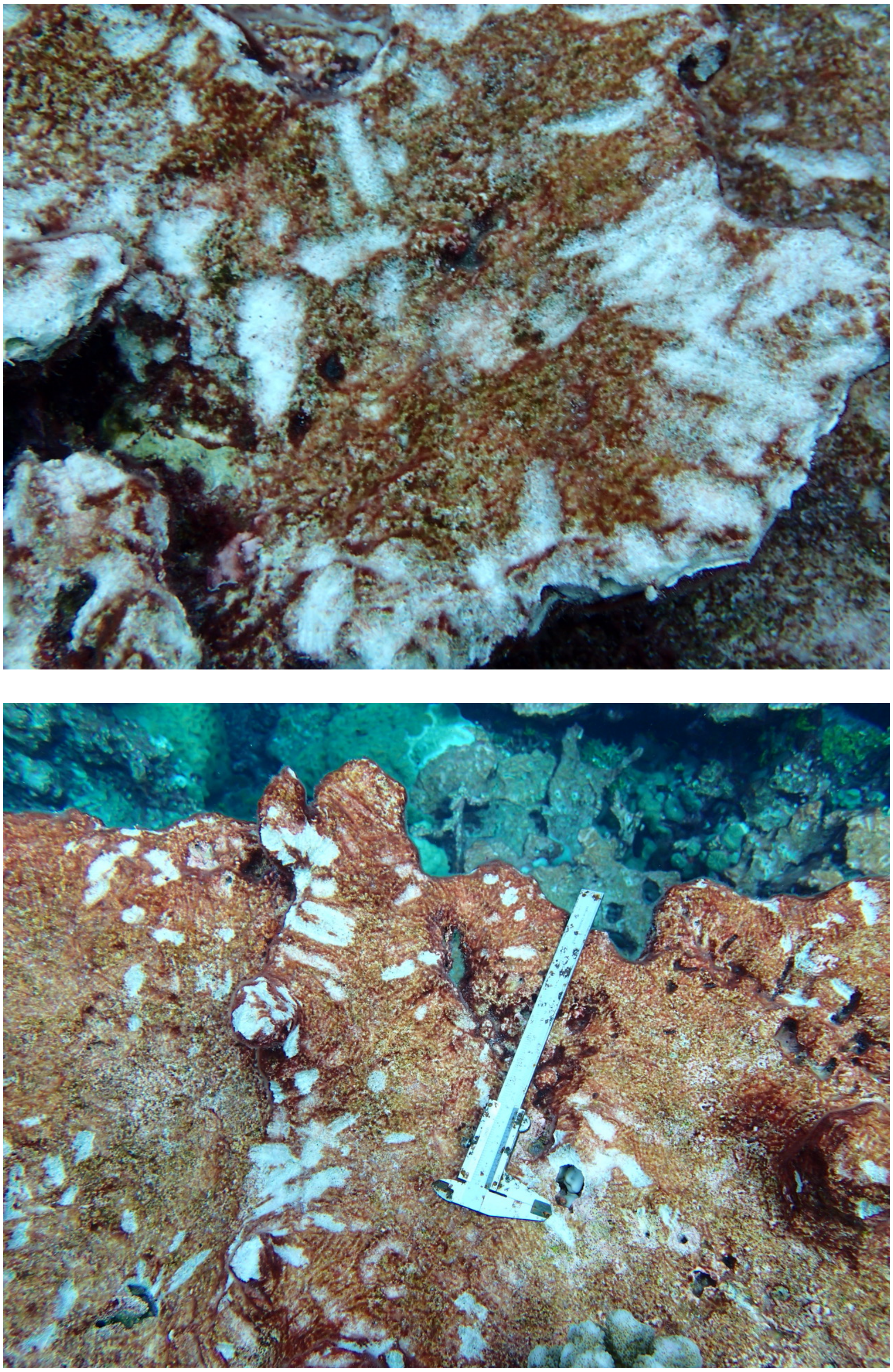

Fig. S3. Photos of natural divots created by parrotfish on Palmyra. The caliper demonstrates the scale/size (Photo Credit: Joe Wible).

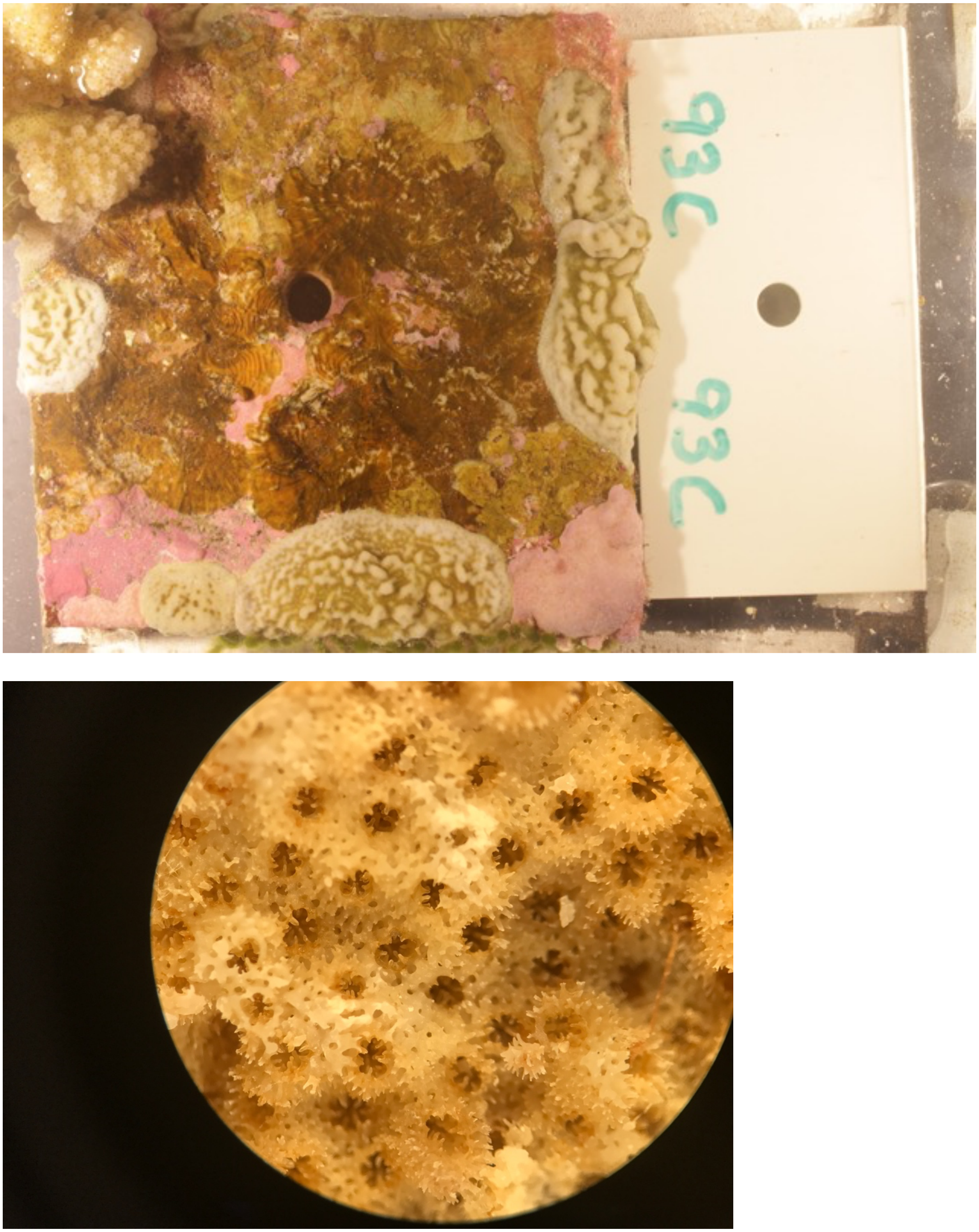

Fig. S4. Photos of coral recruits growing on the settlement tiles after three years.

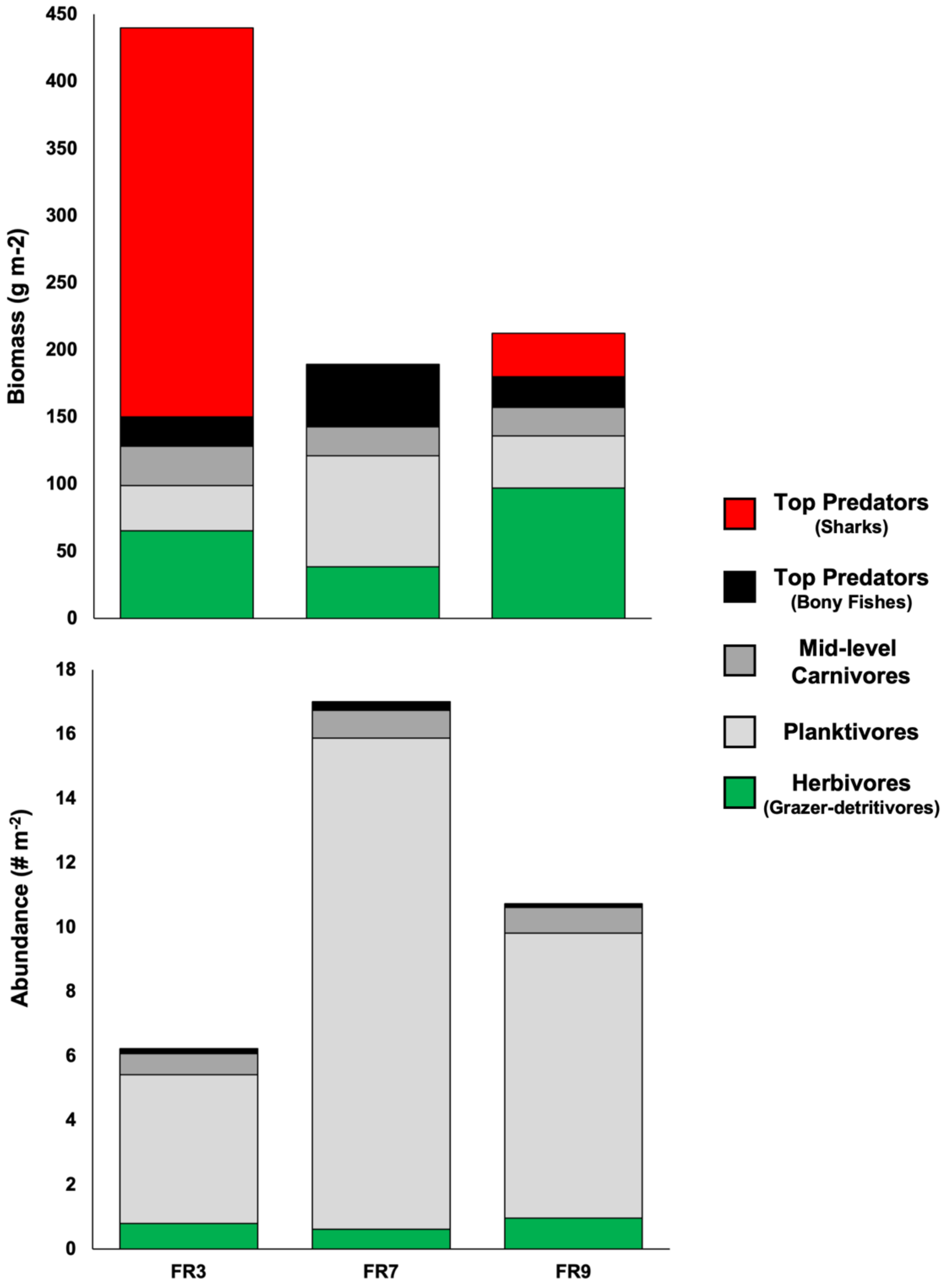

Fig. S5. Average fish biomass and abundance by trophic group across the three sites from surveys conducted in 2014 following methods from (Zgliczynski and Sandin 2017). Data were provided by Dr. Brian Zgliczynski.

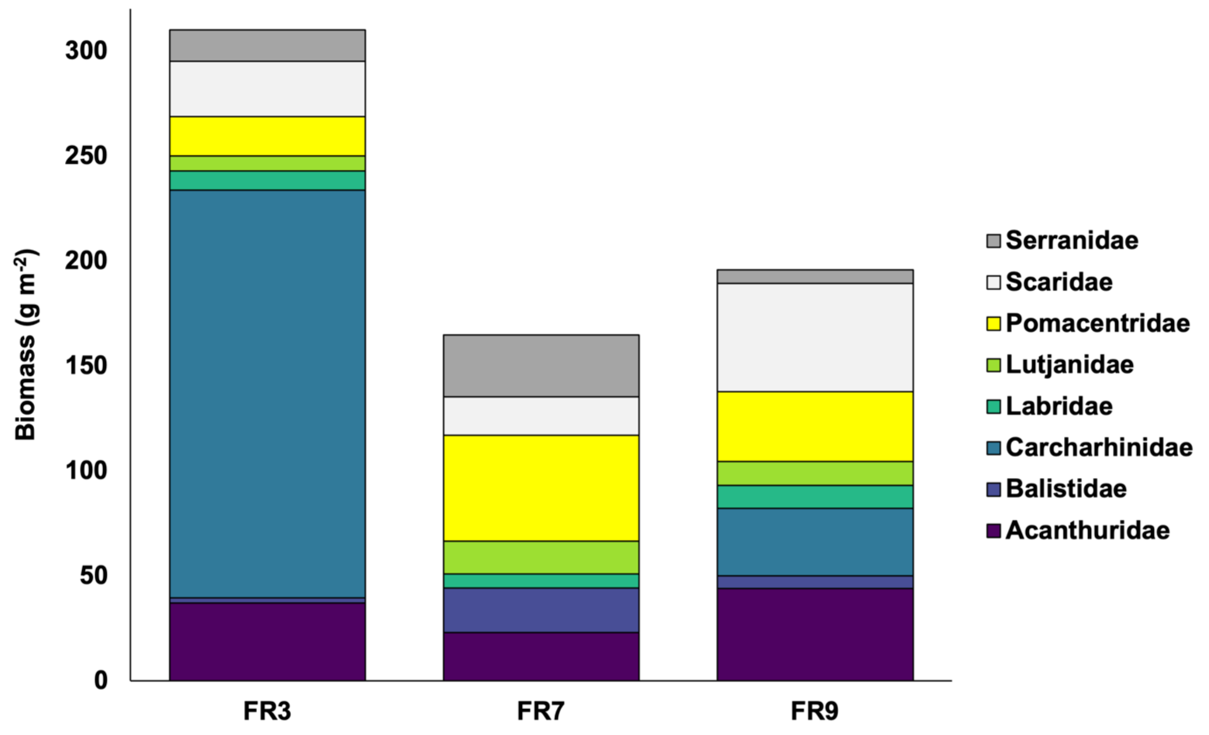

Fig. S6. Average biomass for the top eight families at the three sites during surveys in 2014 following methods from (Zgliczynski and Sandin 2017). Data was provided by Dr. Brian Zgliczynski.

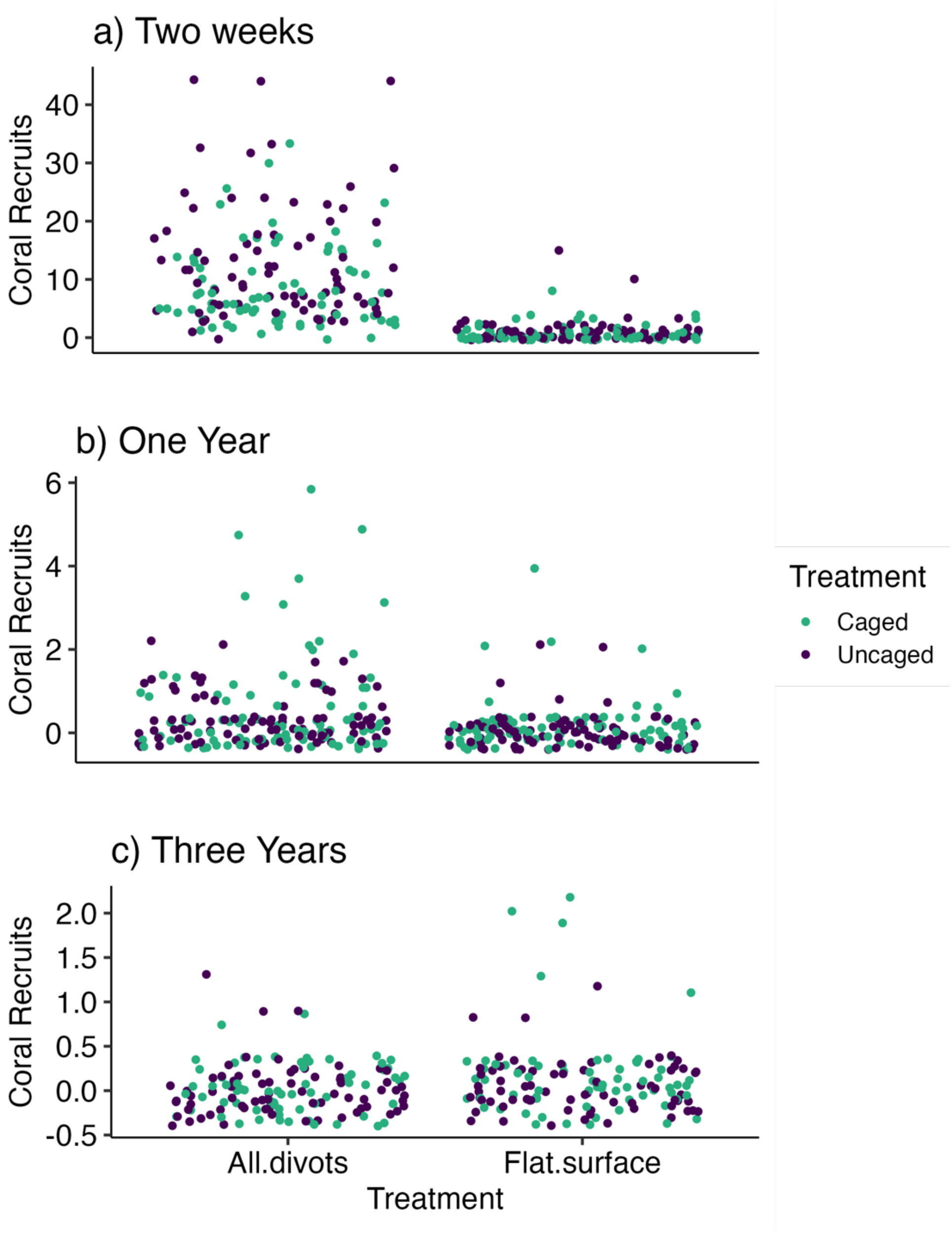

Figure S7. Raw data of coral recruits per each individual tile for divots vs flat surfaces across the two treatments (green = caged, purple= uncaged). Each point represents an individual tile.

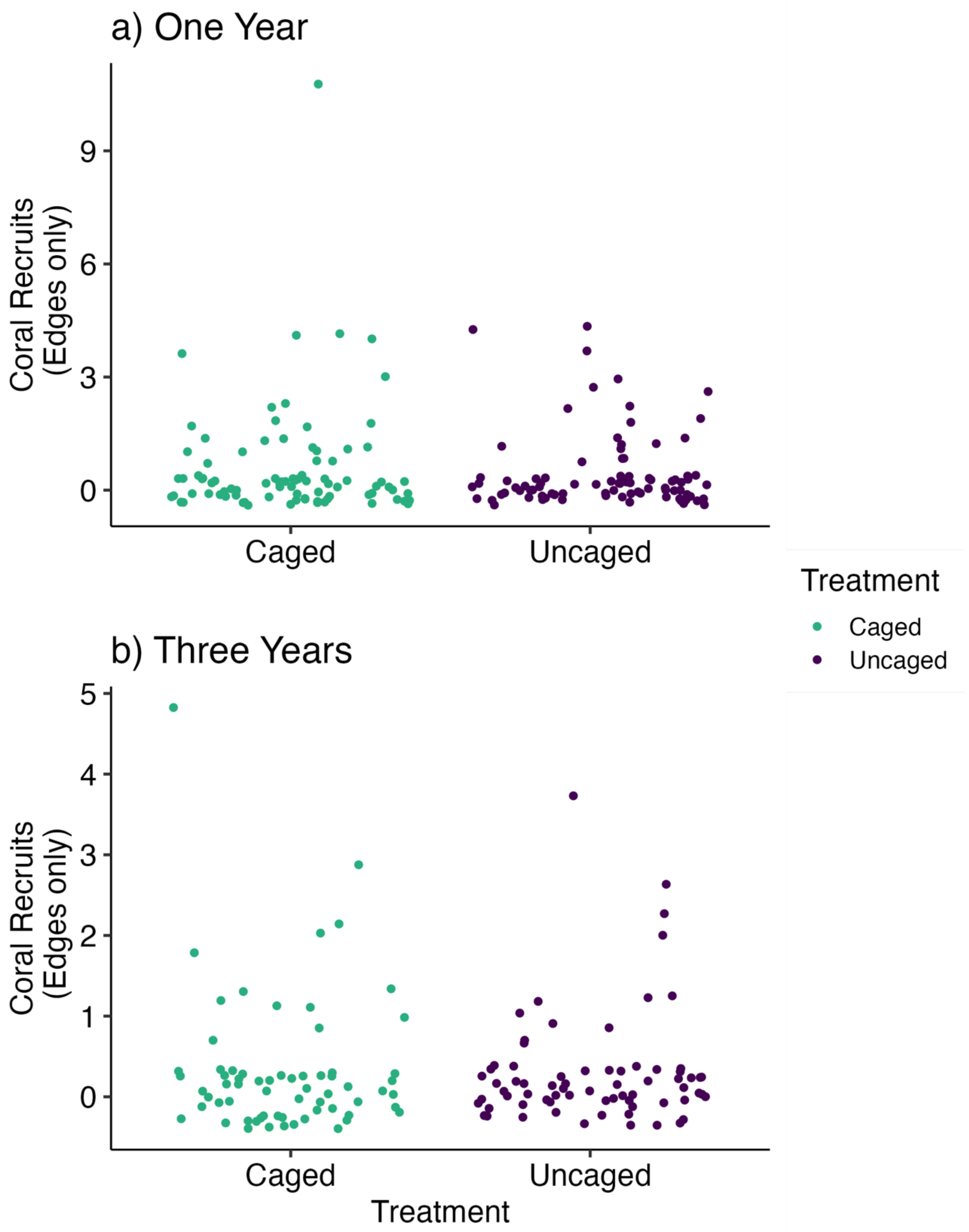

Figure S8. Raw data of coral recruits found on the edges of the tile for the two treatments (green = caged, purple= uncaged). Each point represents an individual tile.

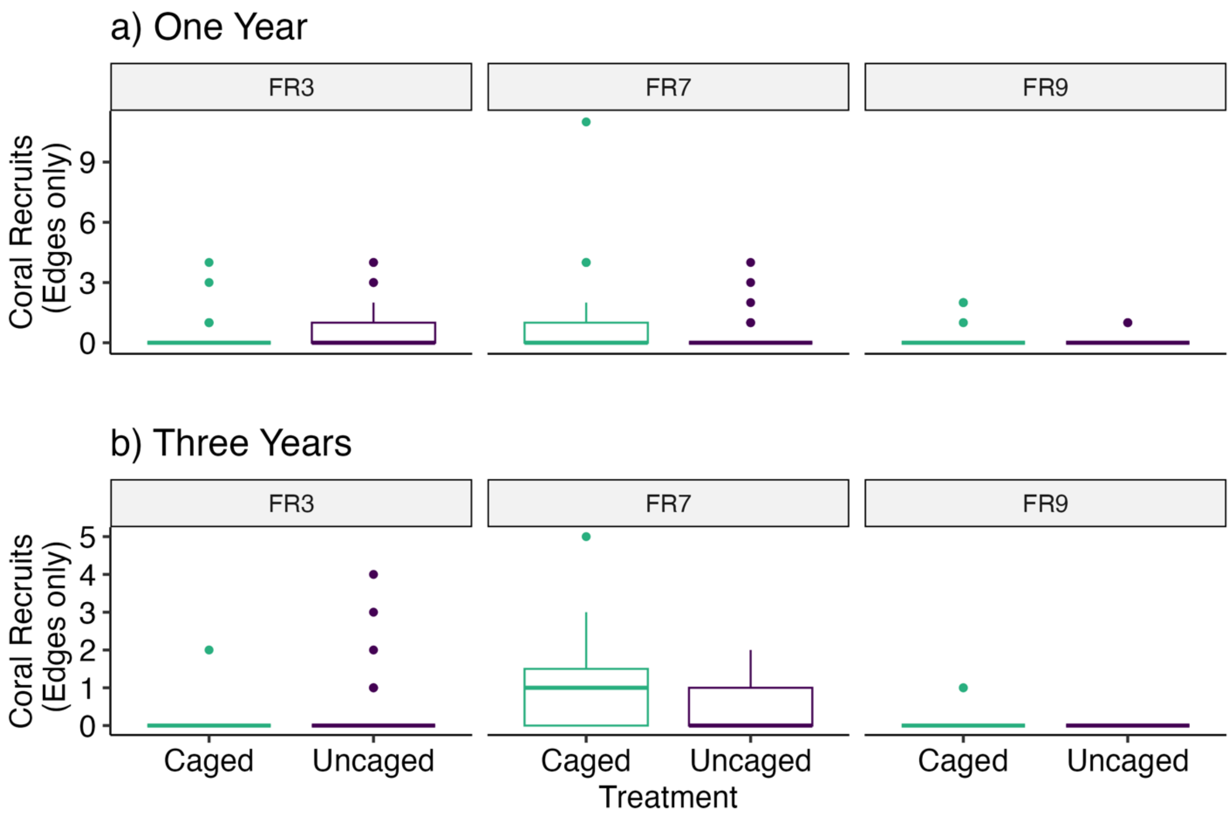

Figure S9. Raw data of coral recruitment on the edges of the tiles by site (FR3, FR7, FR9) for the caged (green) and uncaged (purple) treatments in a) year 1 and b) year 3.

References

Zgliczynski BJ, Sandin SA (2017) Size-structural shifts reveal intensity of exploitation in coral reef fisheries. Ecol Indic 73:411–421
